# PPARs at the crossroads of T cell differentiation and type 1 diabetes

**DOI:** 10.3389/fimmu.2023.1292238

**Published:** 2023-10-20

**Authors:** Farooq Riaz, Ping Wei, Fan Pan

**Affiliations:** ^1^ Shenzhen Institute of Advanced Technology (SIAT), Chinese Academy of Sciences (CAS), Shenzhen, China; ^2^ Department of Otolaryngology, Ministry of Education Key Laboratory of Child Development and Disorders, National Clinical Research Center for Child Health and Disorders (Chongqing), China International Science and Technology Cooperation base of Child Development and Critical Disorders, Children’s Hospital of Chongqing Medical University, Chongqing, China

**Keywords:** type-1 diabetes, peroxisome proliferator-activated receptors, autoimmunity, CD4+ T cells, Th17, regulatory T cells

## Abstract

T-cell-mediated autoimmune type 1 diabetes (T1D) is characterized by the immune-mediated destruction of pancreatic beta cells (β-cells). The increasing prevalence of T1D poses significant challenges to the healthcare system, particularly in countries with struggling economies. This review paper highlights the multifaceted roles of Peroxisome Proliferator-Activated Receptors (PPARs) in the context of T1D, shedding light on their potential as regulators of immune responses and β-cell biology. Recent research has elucidated the intricate interplay between CD4+ T cell subsets, such as Tregs and Th17, in developing autoimmune diseases like T1D. Th17 cells drive inflammation, while Tregs exert immunosuppressive functions, highlighting the delicate balance crucial for immune homeostasis. Immunotherapy has shown promise in reinstating self-tolerance and restricting the destruction of autoimmune responses, but further investigations are required to refine these therapeutic strategies. Intriguingly, PPARs, initially recognized for their role in lipid metabolism, have emerged as potent modulators of inflammation in autoimmune diseases, particularly in T1D. Although evidence suggests that PPARs affect the β-cell function, their influence on T-cell responses and their potential impact on T1D remains largely unexplored. It was noted that PPARα is involved in restricting the transcription of IL17A and enhancing the expression of Foxp3 by minimizing its proteasomal degradation. Thus, antagonizing PPARs may exert beneficial effects in regulating the differentiation of CD4+ T cells and preventing T1D. Therefore, this review advocates for comprehensive investigations to delineate the precise roles of PPARs in T1D pathogenesis, offering innovative therapeutic avenues that target both the immune system and pancreatic function. This review paper seeks to bridge the knowledge gap between PPARs, immune responses, and T1D, providing insights that may revolutionize the treatment landscape for this autoimmune disorder. Moreover, further studies involving PPAR agonists in non-obese diabetic (NOD) mice hold promise for developing novel T1D therapies.

## Introduction

Diabetes is regarded as the most critical and chronic medical condition that is illustrated by the increased blood glucose levels in association with impaired insulin activity due to the defective pancreatic beta cells (β-cell) function (1, 2). As per the definition and classification by the American Diabetes Association, diabetes has four distinct subtypes: (i) Type 1 diabetes (T1D) – an autoimmune disorder marked by insufficient insulin production that extinguishes β-cells; (ii) Type 2 diabetes (T2D) – associated with insulin resistance and a gradual decline in insulin levels accompanied by the loss of β-cells; (iii) Specific type of diabetes ascending from diverse underlying causes; and (iv) Gestational Diabetes Mellitus (GDM) – occurring in the later trimesters of pregnancy, without prior existence before conception (3). It has been estimated that diabetes impacts 9.3% of the overall global population (4), with 19.3% in elderly patients (5). Alarmingly, diabetes is escalating within certain nations (6–9).

Type 1 diabetes (T1D) is a chronic and progressive autoimmune illness. It is a long-lasting disease marked by the body’s incapability to generate insulin. Insulin, produced by β-cells, is a pivotal anabolic hormone that exerts distinct effects on protein, lipid, glucose, and growth (10, 11). T1D primarily occurs due to the death of β-cells in response to recurrent autoimmunity (12, 13). Consequently, T1D emerges as a systemic disorder defined by the hallmark trait of hyperglycemia (14). An array of investigations has underscored the substantial role of genetic, social, economic, and environmental factors in triggering autoimmune responses and ultimately driving the onset of T1D (15–21).

The immune mechanisms driving the autoimmune assault on β-cells have primarily been elucidated through studies conducted in T1D models in rodents (22). Ample evidence indicates that humoral immune responses are pivotal in producing autoantibodies that target the pancreatic β-cells (23, 24). These autoantibodies, including those against insulin, glutamic acid decarboxylase (GAD), islet cell antigens, insulinoma-associated antigen-2 (IA-2), and zinc transporter 8 (ZnT8), initiate an autoimmune response (23, 25–27). This misguided immune attack leads to the progressive destruction of insulin-producing β-cells, resulting in insulin deficiency and elevated blood sugar levels. Also, the presence of these autoantibodies is a hallmark of T1D and is often utilized in precision diagnosis (28, 29). Besides, this autoimmune process occurs when the immune system, primarily coordinated by T cells, initiates an abnormal assault on the insulin-producing β-cells within Langerhans’ pancreatic islets (30). Both CD4+ and CD8+ T cells play pivotal roles in T1D, as their significance in the development of T1D is supported by substantial evidence (31, 32). Meanwhile, it has been understood that specific major histocompatibility complex (MHC) class II haplotypes, and to a lesser extent, MHC class I haplotypes, are associated with an increased predisposition to the development of diabetes (32, 33).

Considering the impact of T cells and β-cells in the pathogenesis of T1D, the present therapeutic strategies have predominantly resolved on either overturning the ongoing immune assault or activating the regeneration of beta cells; however, the effectiveness of these therapies is limited (34, 35). Consequently, a persistent demand exists for approaches that simultaneously attenuate the immune response while enhancing β-cells function. The PPAR family stands out as a promising focus for addressing T1D using this approach because PPARs exhibit anti-inflammatory characteristics, influence the biology of β-cells, and control the lipid composition in the pancreas (36–39). This review aims to provide a comprehensive summary of the current understanding regarding the role of T cells in the pathogenesis of autoimmune T1D and how PPARs play essential roles in mediating the immune responses within the pathophysiological context of T1D. By elucidating the connections between T cell-mediated autoimmune T1D and the modulatory functions of PPARs, this review underscores the potential attractiveness of PPARs as targets for therapeutic interventions in T1D management.

## Role of T cells in the pathogenesis of T1D

The concept of a connection between the immune system and T1D was first introduced in 1973 when researchers discovered a clear connection between HLA antigens and insulin-dependent diabetes mellitus and distinguished it from insulin-independent diabetes (40). Subsequent genome-wide association investigations have established this link and revealed that HLA genes subsidize up to 50% of the genetic susceptibility to T1D, particularly the HLA class II loci. This finding strongly advocates that the selective exhibition of specific autoantigen peptides exerts a critical part in the pathogenesis of T1D (41–43). In the meantime, several meta-analyses have brought to light non-HLA high-risk genetic variations within specific genes, including but not limited to IL2RA, CTLA4, PTPN22, and INS-VNTR (variable number of tandem repeats) (44–47). Studies examining the longitudinal levels of plasma oxylipins and their connection to the risk of T1D in at-risk children showed that higher levels of certain oxylipins related to linoleic acid and alpha-linolenic acid were associated with a reduced risk of T1D (48, 49). These oxylipins, which have pro-resolving and pro-inflammatory properties, may reflect resilience to environmental triggers (50). Conversely, oxylipins related to arachidonic acid (ARA) were linked to an increased risk of T1D, possibly indicating inflammation after the onset of islet autoimmunity (48). Meanwhile, the SNP rs143070873 was strongly linked to the LA-related oxylipin 9-HODE, and rs6444933 (linked with CLDN11) was associated with the LA-related oxylipin 13 S-HODE. Additionally, a locus between LOC100131146 and MIR1302-7, rs10118380 and an intronic variant in TRPM3 were connected to the ARA-related oxylipin 11-HETE, highlighting their involvement in inflammatory signaling and oxylipin production (51). These genetic variations have been associated with a reduced ability to maintain both peripheral and central immune tolerance toward self-antigens and heightened T-cell stimulation and proliferation. These observations underscore the significant role that T cells play in the intricate process of T1D.

**Figure 1:**
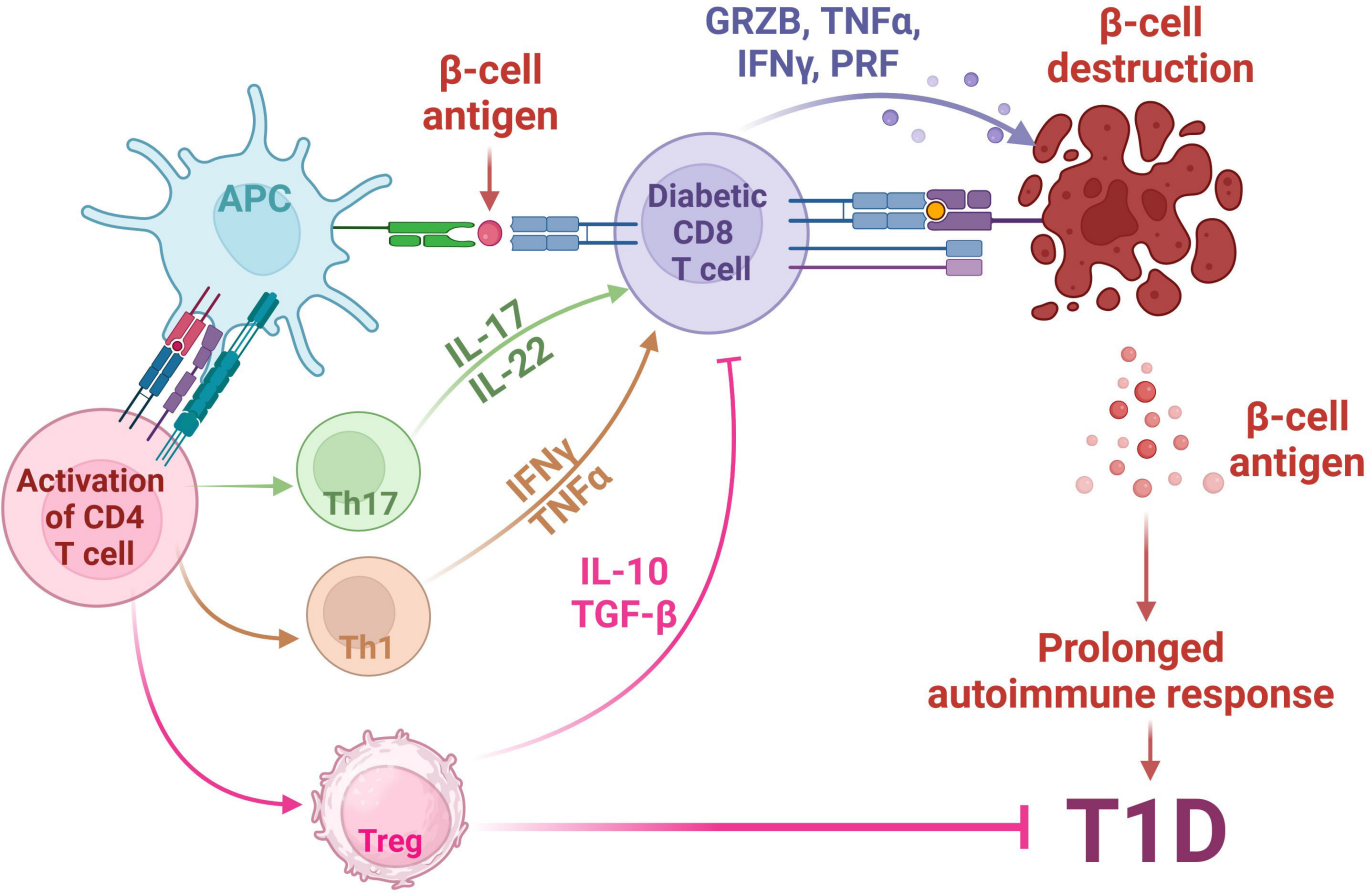
Involvement of T cells in the pathogenesis of autoimmune T1D. The pathogenesis of Type 1 Diabetes (T1D) involves a complex interplay between genetic susceptibility and environmental factors. In individuals predisposed to T1D, environmental triggers lead to stress in pancreatic β-cells, releasing β-cell antigens. These antigens are processed by Antigen-Presenting Cells (APCs) and presented via HLA class II MHC molecules to naïve CD4+ T cells. Activated CD4+ T cells shift towards (i) Th1 phenotype, releasing cytokines such as TNFα and INFγ; (ii) Th17 phenotype, releasing inflammatory cytokines such as IL17 and IL-22; and (iii) Immunosuppressive regulatory T cells (Tregs), which secret anti-inflammatory IL-10 and TGFβ. The activation of Th1 and Th17 activates auto-reactive cytotoxic CD8+ T cells, which then migrate to pancreatic islets, releasing cytotoxic agents, such as granzyme B (GRZB), perforin (PRF), TNFα and INFγ, ultimately leading to the destruction of β cells, a central event in T1D pathogenesis. Meanwhile, the release of anti-inflammatory IL-10 and TGFβ molecules from Tregs inhibits the auto-reactive CD8+ T cell activity, leading to the inhibition of T1D. Created with BioRender.com.

The phenotype of human T1D is recapitulated in the non-obese diabetic (NOD) mouse (52). These NOD mice comprehensively enhanced our knowledge of the T1D pathogenesis. Research conducted in NOD mice has elucidated that the development of T1D depends on the involvement of CD8+ and CD4+ T cells (53). The involvement of T cells in the progression of T1D has been shown in **Figure 1**. Notably, T1D can be transferred solely to immunocompromised syngeneic recipients when splenic T cells, CD8+ and CD4+ T cells, are transferred from a donor NOD mice (53). Conversely, detecting islet-specific autoreactive CD8+ and CD4+ T cells in insulitis lesions, pancreatic draining lymph nodes, and peripheral blood has furnished compelling findings subsidizing the autoimmune nature of T1D (54–58). This presence of autoreactive T cells points towards an impaired immune response, where central immune tolerance weakening towards self-antigens results in the loss of their immune reactivity for foreign proteins, and this weakening is believed to play a role in the insulin-producing cells for the immune attack directed at self-antigens (59–61).

The elevated population of autoreactive CD8+ T cells (which are abundant for particular resident memory cells) in the pancreas of individuals with T1D suggests a differential peripheral regulation and/or activation in patients with T1D (62) as islet autoreactive T cells exhibit different functions than T cells that guard us from infections and cancer. For instance, it was reported that β-cell-specific CD8+ T cells extinguish the insulin-secreting β-cells, predominantly due to the intrinsic events of β-cells (57, 63–65). It was reviewed that CD8+ T cells can diminish the pancreatic β-cells via MHC class I-regulated cytotoxic mechanisms (66). Even though auto-reactive cells are also found in individuals without autoimmune conditions, the elevated numbers of CD8+ T cells, especially resident memory cells, in the pancreas of T1D individuals suggest distinct immune activity in these patients (54, 67, 68).

It is noteworthy to mention that both CD8+ T cells and CD4+ T cells can secrete various effector cytokines, such as interferon-γ (IFNγ), IL (interleukin)-1β, and tumor necrosis factor-α (TNFα) (69, 70). In T1D, the release of IFNγ by CD4 and CD8 cells may destroy β-cells and islets (69). Meanwhile, IFNγ, along with other cytokines, induces the death receptor FAS (also known as CD95) levels, stimulates the production of chemokines by β-cells and elevates their vulnerability to the autoimmune T1D (70–75). The process of β-cell apoptosis can be triggered by the activation of FAS by activated CD4 T lymphocytes that express the FAS ligand (FASL) (76–78). Additionally, the chemokines produced by β-cells contribute to the recruitment of further mononuclear cells to the site, thereby intensifying the inflammatory response (79–81).

The pathogenesis of T1D is believed to commence when there is a low-level demise of β-cells, leading to the exposure of β-cell antigens. Through MHC class II molecules, these antigens are then taken up, processed, and displayed on the cell surface of antigen-presenting cells (APCs) (82, 83). CD4+ T lymphocytes in the surrounding pancreatic lymph nodes proliferate and develop into auto-reactive CD4+ effector T cells (Teffs) in response to antigen presentation and costimulation by APCs (84–86). Immune cell-derived complement proteins (C3a and C5a), which are activated locally during the contact between T cells and APCs, help further to promote Teff growth and function (87). Within the pancreatic islets, these activated Teffs release an array of cytokines, including IFNγ and IL-2, leading to the recruitment of cytotoxic macrophages and CD8+ T lymphocytes (88, 89). These cytotoxic inflammatory cells ultimately infiltrate the islets and initiate the destruction process known as “insulitis”. β-cell death ensues, partly due to direct perforin/granzyme-mediated toxicity by CD8+ T cells and partly as a result of the release of pro-inflammatory cytokines (IFNγ, TNFα, IL-1β) by CD4+ T cells and macrophages (88, 89). Additionally, chemokines released by injured β-cells promote further recruitment of mononuclear cells, and the subsequent release of additional autoantigens enables the expansion and perpetuation of the autoreactive Teff response (90, 91).

Effector T cell subsets Th17, which produces IL-17A, a prominent pro-inflammatory cytokine, and is primarily recognized for its role in attracting other immune cells to sites of inflammation (92, 93). Numerous investigations on rodent models of diabetes suggest the participation of the Th17 subsets in the development of T1D. For instance, in the NOD, a spontaneous autoimmune diabetes model, IL-17F and IL-17A levels in the islets are associated with insulitis. It was suggested that young mice at a prediabetic stage do not exhibit increased expression of IL-17F or IL-17A in the islets. However, older diabetic mice show increased IL-17F and IL-17A, which coincides with the onset of insulitis (94). Inhibiting Th17 cells has significantly reduced diabetes development effectively (95, 96). In IL-17-depleted NOD mice, there is an adjournment in the commencement of diabetes, and insulitis is reduced (97). Additionally, in streptozotocin-induced diabetes, IL-23 plays a role in promoting diabetes development, mainly when subdiabetogenic doses of streptozotocin are administered, as it leads to the expansion of Th17 cells (98). In parallel, clinical T1D patients also support the pathogenic role of Th17. For instance, CD4+ T cells from newly diagnosed T1D adolescents are reported to produce enhanced levels of IL-17 and IL-22 (99). Interestingly, there is no noticeable increase in IFNγ levels or T-bet levels in T1D patients, indicating a bias toward a Th17 response in these individuals (99). Moreover, memory CD4+ T cells from the majority of the T1D patients display elevated secretion of IL-17 and IL-22, signifying an active Th17 response *in vivo* (99). Similarly, alternative investigation detected enhanced IL-17-producing CD4+ T cells in newly diagnosed T1D children (100). Notably, the circulatory CD4+ T cells in T1D patients secrete IL-17 upon activation by β-cell autoantigens (101). A proinflammatory cytokine environment that encourages Th17 development could be accountable for the elevated levels of IL-17 in T1D. In fact, monocytes from individuals with T1D dynamically express considerably more IL-6 and IL-1, encouraging memory CD4+ T cells to produce IL-17 (102).

Besides Teff, the population of CD4+ Foxp3+ regulatory T cells (Tregs) is also impaired, particularly in inflamed islets (103, 104). Foxp3+ Treg cells immunosuppressive cells, which maintain immune balance and modulate self-antigen response (105). The disruption of Foxp3+ Treg homeostasis is believed to enable the favorable differentiation and proliferation of pathogenic β cell-specific Teff (106–108). Though a few studies have concluded that the population of Tregs in the blood of T1D subjects is predominantly unbothered, the analysis of the suppressor function of Tregs isolated from T1D patients shows abridged Treg activity (109–112). Meanwhile, research involving NOD mice has highlighted the significance of Treg cells in prohibiting T1D. Notably, CD28-deficient NOD mice lack Tregs and experience an accelerated disease progression [17]. Another study showed that expression of CD226 in Tregs may lead to decreased Treg immunosuppressive function. In an attempt to elucidate the effect of CD226 in Treg, they found that specific deletion or inhibition of CD226 in Treg enhances the immunosuppressive function of Tregs, which decreases the susceptibility of T1D progression in NOD mice (106). Furthermore, approaches like administering IL-2 to augment Tregs numbers are considered a potential therapeutic avenue in diabetes (113). Although the function of T cells in autoimmune diseases, especially T1D, has been studied widely, further understanding of the role of T cells in T1D pathogenesis is necessary to develop innovative drugs that could enhance the function and population of β-cells by targeting these auto-reactive T cells and elevate the population of Tregs.

## Role of PPARs in β-cells

PPARs, or peroxisome proliferator-activated receptors, were discovered in the 1990s for their role in peroxisome proliferation (114). These receptors belong to the ligand-responsive nuclear hormone receptor family. They include three isoforms in mammals: PPARα, PPARβ/δ, and PPARγ. They primarily regulate lipid metabolism genes, encompassing lipogenesis, lipid storage and transport, and fatty acid oxidation (FAO) (114). These ligand-activated transcription factors are essential in regulating inflammation, energy homeostasis, and addressing issues like obesity and metabolic syndrome (115–117). They form heterodimers with the nuclear receptor RXR, bind to specific DNA-response elements in target gene promoters, recruit coactivators and facilitate chromatin remodeling to initiate DNA transcription (118, 119). Several peptides targeting PPARα (e.g., clofibrate, bezafibrate, fenofibrate) and PPARγ (e.g., ciglitazone, pioglitazone, rosiglitazone, troglitazone) have been employed to mdeicate metabolic conditions like T2D and hyperlipidemia (120–122).

PPAR isoforms exhibit significant functional and structural similarity, yet their expression patterns vary considerably. For instance, PPARα is characterized by its high expression in metabolically active tissues, e.g., adipose tissue, kidney, and liver. It is enhanced during periods of fasting and plays a pivotal role in regulating various metabolic processes. These processes include facilitating fatty acid oxidation (FAO), overseeing amino acid catabolism, modulating lipoprotein metabolism, regulating gluconeogenesis, controlling ketogenesis, and managing inflammatory responses (123, 124). PPARβ/δ, on the other hand, is ubiquitously expressed throughout the body. Its primary role revolves around promoting fatty acid oxidation (FAO). Meanwhile, the activation of PPARβ/δ employs an anti-inflammatory role, leading to abridged production of proinflammatory cytokines, contributing important roles in immune regulation (125, 126). On the contrary, PPARγ demonstrates a broader tissue distribution, with expression observed in numerous organs, including kidney, adipose tissue, intestine, and liver (127, 128). Its functions encompass the modulation of fat cell differentiation, the management of lipid storage, and the facilitation of monocyte differentiation into macrophages. In essence, PPARγ serves an essential role in controlling processes related to lipid metabolism and immune response modulation (129, 130).

In the pancreas, all three PPAR isoforms—PPARα, PPARγ, and PPARδ—are expressed in pancreatic β-cells. It has been believed that PPARα regulates fatty acid oxidation, whereas its expression is influenced by glucose levels (131, 132). Notably, high glucose levels repress PPARα expression in INS-1E cells (islets β-cell line) and primary rat islets (133, 134). Moreover, the glucose-dependent increase in insulin level appears to depend on PPARα, as glucose fails to enhance insulin levels in islets PPARα knockout mice (135). PPARα directly or indirectly impacts the key genes tangled in regulating β-cell function and development. For instance, In INS-1 cells and isolated rat islets, PPAR has been found to increase Pdx-1 levels [a transcription factor important for pancreatic and beta-cell development (136–138)]. Additionally, it was observed that PPARα knockout reduced the level of insulin, MafA [a regulator of insulin secretion (139)], Nkx6.1 [a transcription factor essential for maintaining mature β-cell function (140)], GLUT2, and glucokinase (141).

Interestingly, it was explored that PPARγ directs fatty acids toward esterification (132). Additionally, research investigating the effects of PPAR stimulation or upregulation on insulin secretion and proinsulin production has produced inconsistent findings (142–146). On the one hand, some research has shown that overexpressing PPARγ in INS-1E cells impairs glucose-stimulated insulin secretion (GSIS) (142). On the other hand, several investigations have shown that activation or upregulation of PPAR promotes GSIS in isolated islets and β-cell (147–149). Recently, it has been found that polymorphisms in the IGF1 and PPARγ genes are linked to decreased estimated glomerular filtration rates in children and adolescents with T1D, elevating their susceptibility to early renal complications and impacting the immune response (150–152).

Like PPARα, PPARγ exerts a role in regulating several critical proteins intricately regulating the function and development of β-cells. Activation of PPARγ through compounds like troglitazone (a PPARγ agonist) leads to upregulating genes such as GLUT2, glucokinase, Nkx6.1, and Pdx-1 (153, 154). Furthermore, in PPARγ pancreatic knockout mice, decreased levels of Pdx-1 protein were observed in islets (155). These findings are supported by the presence of peroxisome proliferator response elements (PPRE) sequences in the promoter regions of genes such as Pdx-1 (153, 155), GLUT2 (156), and glucokinase (157).

Although PPARδ is the most prevalent isoform in the pancreas, its impacts on fatty acid oxidation (FAO) have received little attention until recently. PPARδ activation boosts FAO more than PPARα activation does. Fatty acids in the pancreas can cause lipotoxicity and GSIS, which are both long-term impacts on insulin production (149, 158). PPARβ/δ seems to play a crucial part in pancreatic development, as evidenced by studies involving pancreatic PPARβ/δ knockout mice. These mice showed a substantial increase in the mass of β-cells and pancreatic islets (159). This elevation in β-cell mass was connected to higher plasma insulin concentrations, which led to hypoglycemia and better glucose tolerance, and it raises concerns about the detrimental effects of insulin production in the adult pancreas (159) and suggests the negative role of insulin secretion in the mature pancreas. However, these observations contrast with another study, signifying a different role for PPARβ/δ. According to this study, PPARβ/δ promotes the differentiation of beta cells from stem cells by upregulating Pdx-1 (160). This inconsistency in findings underscores the complexity of PPARβ/δ’s role in pancreatic function and development, indicating that its effects may be context-dependent and influenced by various factors. Meanwhile, it is evident that polymorphisms in the promoter region of PPARβ/δ and PPARγ subsidize the genetic susceptibility to T1D and impact the disease score of autoimmunity in islets (151). The impact of PPAR isoforms on the islets suggests that PPAR may exert crucial roles in regulating the function and biology of β-cells (161–163), and further investigations are necessary to explore the part of PPARs in β-cells and T1D.

## Role of PPARs in T cells differentiation

The pathogenicity of T1D includes the intricate interactions of β-cells with various immune cells, particularly T cells (164, 165). The effect of PPARs in T cell regulation and differentiation is multifaceted and characterized by isoform-specific variations. Tregs derived from PPARα knockout mice manifest impaired suppression of CD8+ and CD4+ T cells, diminished migratory capabilities, and reduced expression of several chemokine receptors (166). This phenomenon aligns with the prolonged inflammatory response observed in PPARα knockout mice upon exposure to agents like arachidonic acid (167). In mouse models, the Fenofibrate, a PPARα agonist, has been shown to elevate Foxp3+ regulatory T cells (168, 169). Similarly, our recent study emphasizes that knockout of PPARα in mice diminishes the population of Th17 cells, whereas treating T cells with fenofibrate elevates the population of Th17 (170). Mechanistically, we found that PPARα-depletion augments the activity of IKKα, which positively contributes to the transcription of IL17A by interacting with RORγ. Meanwhile, IKKα also interacts with Foxp3 for its proteasomal degradation, thus leading to an elevated population of Th17 cells. Notably, PPARα ablation augmented the IL-17+Foxp3+ double-positive cells in the brain of the EAE-induced animal model (170). These results indicate that activation of PPAR may inhibit the formation of Th17 cells and enhance the percentage of Foxp3+ Tregs, which may slow the evolution of autoimmune disorders, including T1D.

PPARα also regulates effector T cells, with heightened PPARα expression associated with augmented secretion of Th2-related cytokines. Conversely, PPARα knockout mice exhibit a greater propensity for differentiation toward a Th1 phenotype (171). The PPARα agonist WY14643 has also been observed to curtail proliferation in human T cells and enhance depletion of T cells by arresting them in the G2/S phase (172). Patients with hyperlipidemia who undergo fenofibrate treatment experience reduced levels of IFNγ and TNFα (173). These findings are corroborated by PPARα knockout mice observations, where elevated TNFα and IFNγ levels are evident (171).

The role of PPARγ in regulating the ratio of regulatory to effector T cells is now becoming clear. In PPARγ knockout mice, decreased PPARγ activity is correlated with a higher number of effector T cells, which is distinguished by increased antigen-specific proliferation and excessive IFNγ production in response to IL-12 (174). Furthermore, it has been demonstrated that PPAR inhibits RORt expression, preventing the development of Th17 cells in both humans and mice (175). In mouse models of colitis, PPARγ agonists, such as troglitazone and rosiglitazone, have been observed to alter the immune response from Th1 to Th2, resulting in reduced Th1-related transcription factors, cytokines, chemokines, and heightened expression of Th2-associated factors (176, 177). Conversely, PPARγ deficiency is associated with a diminished CD4+ Foxp3+ regulatory T cell population (178). This is underscored by identifying a specific Treg subset characterized by high PPARγ expression within visceral adipose tissue (179). PPARγ serves a central role in orchestrating these Tregs, as evidenced by the Treg formation prevention upon Treg-specific PPARγ deletion. Additionally, PPARγ activation encourages the growth of Foxp3+ regulatory T cells, whereas PPARγ depletion in Tregs increases the responses of effector T cells (174, 178, 180). Thus, the impact of PPARγ on Tregs may be context-dependent. Notably, PPAR-γ is essential in elevating the differentiation of Tregs and regulating insulin resistance. This occurs through a synergistic mechanism that reduces the expression of pro-inflammatory cytokines such as IL-6, TNFα, and IL-1β, while simultaneously enhances the anti-inflammatory cytokines like TGF-β, and IL-10 (181). In addition to the role of PPARs in the differentiation of Th1, Th2, Treg, and Th17 subsets, PPARγ likely to influence the generation of follicular helper T cells (Tfh). It was investigated that mice with CD4 cell-specific PPARγ knockout exhibit increased Tfh cell activation and a greater propensity for germinal center formation (182).

Activation of PPARβ/δ inhibits Th17 and Th1 responses while bolstering Th2 responses (183–185). In contrast, deletion of PPARβ/δ produces an opposing outcome. This discrepancy can be attributed to PPARβ/δ’s role in promoting FAO, thereby preventing the T cell proliferative burst that occurs after antigen identification as metabolism shifts from oxidative pathways to glycolysis (186–188). Although the information regarding the role of PPARs in autoimmune diseases is limited, the available data suggests that activation of PPARs, especially PPARα, may restrict the development of autoimmune diseases.

## Therapeutic potential to target PPAR in T1D

Considering the importance of herbal medicines (189, 190), numerous researches have been conducted to find herbs and natural compounds for treating T1D (191–193). In addition, a few studies also investigated various natural compounds that exhibited potency to target PPARs and have the potency to cure T1D. For instance, it has been studied that Astilbin, a flavonoid compound initially discovered for its ability to suppress effector CD4+ T cells by inhibiting their function (194), activates the ROS-dependent PPARγ pathway which leads to the suppression of effector CD4+ T cell activities through direct binding to Cytochrome P450 1B1. Consequently, It was suggested that Astilbin exerts immune-suppressive effects by downregulating the secretion of inflammatory cytokines by CD4+ T cells in the NOD mice (195). Similarly, another flavonoid, Epigallocatechin gallate, also decreases the progression of T1D by activating PPAR-γ (196–198). Curcumin, a PPAR agonist, has shown the ability to anticipate the damage of islets by exerting a protective impact on the β-cells (199, 200). A recent study exhibits that Curcumin exerts protective effects on the autoimmune T1D (201). They found that Curcumin dampens T lymphocyte responses by inhibiting proliferation and production of IFNγ, affecting the T-bet transcription factor. It also reduces NF-κB activation in NOD lymphocytes stimulated via TCR (201, 202).

In the pancreas, activation of PPAR enhances fatty acid oxidation, which can acutely potentiate GSIS. The PPARγ-agonist pioglitazone was observed to boost GSIS in db/db mice while the PPARα-agonist fenofibrate inhibited GSIS in newborn rats with active obesity (203, 204). This discrepancy may be attributed to the minimal level of PPAR-γ in INS-1E cells. In those with recently discovered T1D, pancreatic islets exhibit reduced sulfatide levels (23% of those in control participants) and decreased sphingolipid metabolism-related enzyme levels. Fenofibrate, known to activate sulfatide biosynthesis and act as an anti-inflammatory drug (205), ultimately impeded T1D in NOD mice (151). In a 19-year-old female with newly diagnosed T1D, fenofibrate medication started seven days after diagnosis disregarded the need for insulin therapy (206).

Numerous PPAR antagonists have been synthesized, although not initially developed for diabetes treatment (207). For example, a synthetic potent PPAR-α antagonist, GW6471, is primarily employed as a pharmacological tool for identifying effects that are reliant or independent of PPARs. GW9662, which has been elucidated as a PPAR-γ antagonist, facilitates the recruitment of NCOR1 nuclear receptor corepressor 1 (NCoR). Additionally, GSK3787 and GSK0660 serve as PPAR-δ antagonists for pharmacological purposes. Notably, GSK0660, when employed solely in human retinal microvascular endothelial cells, exhibits inverse agonist activity, inhibiting the TNFα-dependent level of numerous chemokines (208, 209). Similarly in the brain, agonists for PPAR-γ (rosiglitazone), PPAR-δ (GW501516), and PPAR-α (fenofibrate), as well as their respective antagonists (GW9662, GSK0660, and GW6471), collectively reduce the production of the pro-inflammatory cytokine TNFα in rat astrocytes under the influence of lipopolysaccharide (LPS) (210).

## Conclusion

In conclusion, the cumulative prevalence of Type 1 diabetes (T1D), particularly in Asian countries, presents a significant challenge to healthcare systems due to its associated complications, treatment costs, resource limitations, and low awareness levels. To reduce this burden and mortality due to diabetes, countries must comprehend the extent of the disease and develop effective strategies. Recent research has unveiled the intricate interplay between CD4+ T cell subsets, particularly Th17 cells and Tregs, in autoimmune diseases like T1D. Th17 cells promote inflammation and immune responses, whereas Tregs exert immunosuppressive functions, striking a balance critical for immune homeostasis. The evolving understanding of the pathogenesis and etiology of T1D emphasizes the roles of both adaptive and innate immunity in driving the autoimmune response against pancreatic β-cells. Immunotherapy shows promise in regaining self-tolerance and preventing harmful autoimmune reactions, but further investigations are needed to refine these treatments.

In the realm of molecular mechanisms, PPARs have gained attention not only as regulators of lipid metabolism but also as potent modulators of inflammation and β-cell biology. While their potential in modulating T cell responses and impacting T1D remains largely unexplored, studies on PPAR agonists in NOD mice show promise. Thus, future investigations should focus on unraveling the precise roles of PPARs in T1D pathology, offering a novel treatment approach that targets both the immune system and pancreatic function. Conversely, as the utilization of medicinal plants and their derivatives has shown promise in reducing the overall prevalence of T1D by augmenting the population of Tregs and activating the PPARs, further research should prioritize the extraction of novel herbal plants or the purification of their derivatives for consideration in diabetes treatment. This immunomodulatory effect underscores the potential of herbal remedies in T1D management. Moreover, these promising herbal interventions warrant more extensive exploration through clinical trials, potentially offering novel and effective therapeutic options for individuals living with T1D.

## Author contributions

FR: Conceptualization, Writing – original draft, Writing – review & editing. PW: Writing – original draft, Writing – review & editing. FP: Conceptualization, Writing – review & editing.
